# Plant Antimicrobial Agents and Their Effects on Plant and Human Pathogens

**DOI:** 10.3390/ijms10083400

**Published:** 2009-07-31

**Authors:** Rocío González-Lamothe, Gabriel Mitchell, Mariza Gattuso, Moussa S. Diarra, François Malouin, Kamal Bouarab

**Affiliations:** 1 Département de Biologie, Faculté de Sciences, Université de Sherbrooke, 2500 Boulevard de l’Université, Sherbrooke, Québec J1K 2R1, Canada; E-Mails: Rocio.Gonzalez@USherbrooke.ca (R.L.); Gabriel.Mitchell@USherbrooke.ca (G.M.); Mariza.Gattuso@USherbrooke.ca (M.G.); 2 Pacific Agri-Food Research Centre, Agriculture and Agri-Food Canada, P.O. Box 1000, Agassiz, BC V0M 1A0, Canada; E-Mail: Moussa.Diarra@agr.gc.ca (M.D.)

**Keywords:** antimicrobial, phytoalexin, phytoanticipin, secondary metabolites, infectious disease, cystic fibrosis, phytoprotection

## Abstract

To protect themselves, plants accumulate an armoury of antimicrobial secondary metabolites. Some metabolites represent constitutive chemical barriers to microbial attack (phytoanticipins) and others inducible antimicrobials (phytoalexins). They are extensively studied as promising plant and human disease-controlling agents. This review discusses the bioactivity of several phytoalexins and phytoanticipins defending plants against fungal and bacterial aggressors and those with antibacterial activities against pathogens affecting humans such as *Pseudomonas aeruginosa* and *Staphylococcus aureus* involved in respiratory infections of cystic fibrosis patients. The utility of plant products as “antibiotic potentiators” and “virulence attenuators” is also described as well as some biotechnological applications in phytoprotection.

## 1. Introduction

Plants are continuously in contact with different microorganisms, including viruses, bacteria and fungi. The relationships established with some of them are beneficial for the plants; thus, some bacteria known as rhizobia, form symbiotic association with leguminous plants by fixing atmospheric nitrogen in root nodules. Other bacteria found close to the plant root (rhizobacteria) are able to control plant diseases caused by soil pathogens [1]. Fungal interactions can also be positive for the plant, stimulating its growth and development as in the case of *mycorrhizae* [2]. But many plant-associated microbes are pathogens that affect plant development, reproduction and ultimately yield production. The control of these pathogens is a major challenge in agriculture.

To arrest the spread of pathogens, plants possess an innate immunity that involves different layers of defence responses. Some of these defences are preformed and others are activated after recognition of pathogen elicitors [3], and include reinforcement of the cell wall, biosynthesis of lytic enzymes and production of secondary metabolites and pathogenesis related proteins [4]. In this review, we will focus on the description of the secondary metabolites, both preformed and pathogen-induced, that the plant accumulates in response to pathogen invasion, with special emphasis on their biological role against microorganisms and their biotechnological values as potential antimicrobials in plant protection and human health.

## 2. Phytoanticipins *versus* Phytoalexins

**Figure 1.**
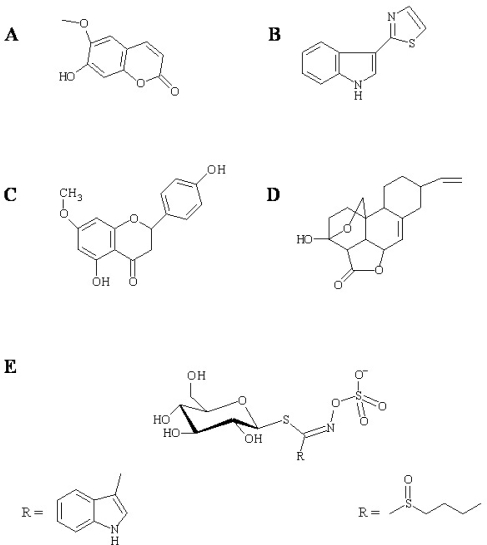
Examples of antimicrobial phytoalexin structures. (A) Scopoletin from tobacco, (B) camalexin from *A. Thaliana*, (C) sakuranetin, (D) nomilactone B from rice, and (E) glucosinolates from *Brassicacea*. Structures of the R groups of indol-3-ylmethyl (E, left) and 4-methylsulfinylbutyl glucosinolate (E, right) are shown as examples of Arabidopsis tryptophan- and methionine-derived glucosinolates, respectively.

The antimicrobial plant compounds that have received more attention in plant defence are the phytoalexins (Figure 1). Phytoalexins are antimicrobial compounds which require *de novo* expression of the enzymes involved in their biosynthetic pathways after elicitation [2]. Therefore, the production of phytoalexins requires transcriptional and/or translational activity in the plant once the pathogen has been detected. The induced response mechanism also involves the trafficking and secretion of antimicrobial compounds to the infection site [5]. This definition of phytoalexins differs from the original one by Müller and Börger [6] and avoids the assignation of a role in disease resistance for these molecules, because, although a function in plant defence is assumed for these compounds, such a role cannot always be easily proven.

**Figure 2.**
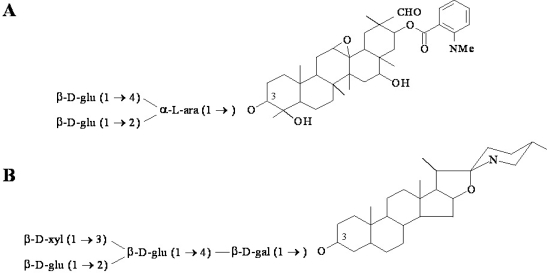
Examples of antimicrobial phytoanticipin structures. (A) The major oat root saponin avenacin A-1, and (B) the saponin α-tomatine from tomato. Tomatidine is the aglycon version of the phytoanticipin tomatine.

Similarly, the term phytoanticipin was coined by vanEtten *et al.* [7] referring to “low molecular weight antimicrobial compounds that are present in plants before challenge by microorganisms or are produced after infection solely from preexisting constituents” (Figure 2). Some phytoanticipins are found at the plant surface. Others are sequestered as preformed compounds in vacuoles or organelles and released through a hydrolyzing enzyme after pathogen challenge. Because the enzyme involved in the final liberation of the molecule is not formed *de novo* these compounds are not considered as phytoalexins [8].

The previous definitions are based on the dynamic of the synthesis of the antimicrobial molecule, not on its chemical composition, which can be confusing sometimes since the same chemical can be a phytoalexins in one plant and a phytoanticipin in another and moreover, the same molecule can be a phytoalexin or a phytoanticipin in different organs of the same plant [2].

Several pieces of evidence indicate that preformed and induced antimicrobial chemicals confer protection against disease. We will focus next on the description of examples of phytoanticipins and phytoalexins for which the biological roles in plant defence responses have been characterized.

### 2.1. Phytoanticipins involved in defence responses

In this section, we describe the role of saponins in plant disease resistance. Saponins are glycosylated phytoanticipins that are found in a wide range of plant species and can be divided into three major groups, triterpenoid, steroid or steroidal glycoalkaloid, depending on the structure of their aglycones [8]. Because they have potent antimicrobial activities it is proposed that the natural role of these molecules in plants is to confer protection against potential pathogens [9]. The saponins studied in deepest detail in relation to their potential role in defence are avenacin and α-tomatine. Avenacins are oat root saponins. The antifugal activity of avenacin is associated with its ability to form complexes with sterols present in fungal membrane leading to pore formation and loss of membrane integrity [10]. The localization of the major avenacin, avenacin A-1, in the epidermal cell layer of oat root tips and in the emerging lateral root initials, suggests a role as a chemical barrier [11]. Moreover, the capacity of *Gaeumannomyces graminis* var *avenae* to detoxify avenacin A-1 has been shown to be essential for its interaction with oat. Fungal mutants lacking the saponin-detoxifying enzyme avenacinase showed increased sensitivity to avenacin A-1 and were no longer able to infect [12]. Saponin-deficient mutants also showed compromised resistance to several pathogens, indicating that avenacins provide a preformed chemical defence to pathogen attack [13]. Interestingly, accumulation of avenacin biosynthesis pathway intermediates in oat roots results in callose accumulation, a well known defence mechanism which suggests that phytoanticipin accumulation may also trigger other defence responses [14]. This implies that the antimicrobials may work in defence-related signalling processes and strengthen the relevance of these compounds as biotechnological weapons against pathogen infection [5].

The major saponin in tomato is α-tomatine. This phytoanticipin is accumulated in healthy plants in its biologically active form. Relationship between α-tomatine accumulation and disease resistance has been difficult to show in tomato, since the tested fungi showed some degree of resistance to the compound by producing tomatine-detoxifying enzymes [8]. Actually, for some phytopathogenic fungi, the production of α-tomatine-detoxifying enzymes is a determinant of virulence against α-tomatine-containing host, providing an evidence of the role of this compound in plant defence. Thus, *Septoria lycopersici* produces tomatinase, an extracellular enzyme that hydrolyses α-tomatine to β_2_-tomatine, which is less toxic to the fungus. Infection with a tomatinase-deficient strain of *S. lycopersici* enhanced defence responses in tomato [15] and the mutant strain was unable to infect the normally susceptible host *Nicotiana benthamiana* [16]. More interestingly, the degradation product of α-tomatine is able to suppress the defence response. Thus, inoculation of *N. benthamiana* with α-tomatine or β_2_-tomatine before infection with the tomatinase-deficient mutant of *S. lycopersici* allows the mutant pathogen to infect the host in the case of β_2_-tomatine but not α-tomatine. Moreover, pretreatment with β_2_-tomatine increases the resistance to *Pseudomonas syringae pv tabaci* conferred by the gene *Pto* (from *Pseudomonas tomato* resistance gene) [16]. Similarly, *Fusarium oxysporum* f. sp *lycopersici* is able to degrade α-tomatine into tomatidine and lycotetraose, compounds that inhibit the hypersensitive response induced cell death in tomato [17]. Therefore in the previous examples, pathogen resistance cannot only be attributed to the disappearance of the antimicrobial compound, but also to the capacity of the degradation product of the phytoanticipins to suppress defence responses. In this case it is difficult to elucidate the role of the phytoanticipins themselves in pathogen resistance, since the fungus has evolved a sophisticated way to overcome defence responses by taking advantage of the degradation product of these molecules [17].

### 2.2. Phytoalexins: Some biological examples

In this section we discuss the role of the tobacco phytoalexin scopoletin, the phytoalexins in the defence response of rice and crucifers and the biological function of phytoalexins found in the root exudate.

The hydroxycoumarin scopoletin (6-methoxy-7-hydroxycoumarin) is the major phytoalexin in tobacco plants. This compound is well known to display antimicrobial properties *in vitro* [18] and accumulates in tobacco reacting to pathogens and elicitors [19,20]. It is responsible for the appearance of a remarkably bright blue fluorescence under UV light in the tissues surrounding necrotic lesions [21] and it has been proposed to act as a scavenger of reactive oxygen species produced in excess after triggering of the hypersensitive response [22]. Interestingly, the reduction of scopoletin and the glucoside form of scopoletin (scopolin) levels in tobacco plants has been associated with a decrease of resistance to infection with TMV [19] providing an evidence of the antimicrobial role of this compound *in planta*.

Rice is among the most economically important world crops. One of the most serious diseases affecting rice is the rice blast produced by *Pyricularia oryzae*, which can cause important yield losses. One approach to improve rice resistance to blast could be the culture of rice genotypes producing anti-*P. oryzae* phytoalexins [2]. Therefore, the analysis of the correlation between rice blast infection and production of host phytoalexins has been an important area of research in the past. There are a wide variety of secondary metabolites produced in rice after elicitation of the host response for which antifungal activity has been shown. Among them sakuranetin and momilactone A have attracted special interests [2]. Sakuranetin is a powerful antifungal derived from the flavonoid naringenin [23] while momilactone A is a diterpenoid [24]. For both of them, an accumulation of the molecule in the disease lesion of rice – *P. oryzae* incompatible interaction (Tetep rice cultivar which is resistant to *P oryzae*) has been shown [25]. Moreover, a correlation between the rice genotypes that accumulate higher concentrations of these phytoalexins and the degree of resistance to *P. oryzae* was established [25]. Nevertheless in these experiments the induction of each of the phytoalexin synthesis was done through exposure to UV light and interpretations from the results have to be taken carefully since differences can be observed between the production of phytoalexins after UV exposure and *P. oryzae* infection. More recently a work showing that enhancing of the momilactone accumulation in rice after silicon treatment increased rice resistance to blast has been published [26]. In sum, these results indicate that phytoalexins can be a determinant molecular weapon against blast pathogen infection in rice.

The plant family *Brassicaceae* (crucifers) includes a large number of economically important crops and their members are known to synthesize a vast variety of secondary metabolites involved not only in defence against microorganisms, but also in human health. Most of the phytoalexins of this family are derived from tryptophan, and they have been involved in protection against biotic and abiotic stresses [27]. Several phytoalexins of the crucifers have been shown to have growth inhibitory activities against fungal pathogens [28], but attempts to show a role of these molecules as pathogen inhibitors *in planta* have been mostly unsuccessful due to the ability of the tested fungi to metabolize the phytoalexins.

Recent studies show that glucosinolates, thioglucosides constitutively stored in crucifers, are also involved in the response to pathogens [29,30]. Glucosinolates and their degradation products have been traditionally considered anti-insect compounds but they also have been proposed as antimicrobials [5]. Recently a metabolic pathway of glucosinolates that differs from the pathway activated by chewing-insect was identified. This new pathway is involved in antifungal defence responses and involves the biosynthesis of the compound 4-methoxyindol-3-ylmethylglucosinolate (4-methoxy-I3G) and subsequent activation by a myrosinase [29]. More interestingly, both the synthesis and the degradation of 4-methoxy-I3G are mandatory for the accumulation of callose after pathogen attack and ultimately resistance to several microbial pathogens [30]. These results suggest that 4-methoxy-I3G degradation products are either cofactors or elicitors of callose deposition, and suggest an additional role for the phytoalexin besides antimicrobial activity [5].

The best studied phytoalexin is camalexin, from the model plant *Arabidopsis thaliana*. Camalexin refers to the molecule 3-thiazol-2’-yl-indole which was isolated from the leaves of the crucifer *Camelina sativa* infected with *Alternaria brassicae* [31]. This phytoalexin has been found in other members of the crucifers, but we will refer here only to studies in *Arabidopsis*, since it provides an exceptional model for the investigation of phytoalexins in defence responses.

Camalexin production is induced in *Arabidopsis* after infection with bacteria, viruses, fungi and oomycetes. As most of the phytoalexins from crucifers, it is synthesized from tryptophan. The first step is the synthesis of indole-3-acetaldoxime (IAOx) catalyzed by CYP79B2 and CYP79B3. The IAOx-metabolizing steps are not well known until the last step of the pathway in which dihydrocamalexic acid is decarboxylated to camalexin [31]. High concentrations of camalexin have been observed at the infection site of *Alternaria alternata* [32] and in the proximity to the lesions induced by *Botrytis* [33], and this distribution correlates with a high induction of tryptophan and camalexin biosynthetic genes [32,34]. We previously mentioned that camalexin production is induced by a high number of pathogen, but a growth inhibition effect of the phytoalexins has been shown only for some of them [31]. Several attempts to correlate *in vitro* antimicrobial activity and plant resistance to infection have been done. An advantage in the use of *Arabidopsis* is the availability of a high number of mutants that allow studying the role of camalexin in the defence response. The mutant *pad3* is unable to metabolize the last step in the synthesis of camalexin, and therefore the molecule is not accumulated in this mutant. The *pad3* mutant did not show an increased sensitivity to infection by *Pseudomona syringae* pv *maculicula* in respect to the control. Moreover, camalexin was shown only to disrupt the integrity of bacterial membranes at a concentration that is probably much higher than the one reached *in planta* and this further suggests that this compound is not necessary to stop the bacterial infection [35]. On the other hand, *pad3* mutants show enhanced susceptibility to *Alternaria brassicicola* and *Leptosphaeria maculans* [35,36], which agrees with a 10-fold lower concentration of the phytoalexin needed to inhibit susceptible fungi compare to Gram negative bacteria [35].

Other works that have not used mutant hosts also suggest a role for camalexin in defence responses. Thus, wounding of *Arabidopsis* leaves have been shown to increase the resistance to *B. cinerea* in a camalexin-dependent way, suggesting a determinant role for this compound in *Arabidopsis* resistance against *B. cinerea* [37]. On the other hand, studies on the virulence of pathogen isolates with different susceptibilities to camalexin have also been carried out for *Botrytis cinerea* [33]. Kliebenstein *et al*., showed that the degree of tolerance of *B. cinerea* isolates to camalexin determines the ability of the isolate to infect the wild-type plant, while they can all infect a camalexin-deficient mutant. Therefore, camalexin contribution to the host defence response is limited to the phytoalexin-susceptible isolates. Interestingly, the camalexin-susceptible pathogens induce much higher accumulation of camalexin than camalexin-tolerant ones. The results show that camalexin is an important defence response in *Arabidopsis* against *B. cinerea* and suggest that some *B. cinerea* isolates are able to overcome this response by an unknown mechanism. For instance, the induction of an ABC transporter that supports efflux of fungitoxic compounds after camalexin exposure was reported for *B. cinerea*. Accordingly, a strain lacking the functional transporter is more susceptible to camalexin *in vitro* and less virulent on wild-type plants, but is still fully virulent on camalexin-deficient mutants [38]. This work describes a virulence factor in *B. cinerea* that allows the pathogen to overcome a plant defence mechanism and strengthen the argument about camalexin being a determinant defence against *B. cinerea*.

Several examples of fungi metabolizing camalexin have been also reported and include virulent isolates of *Rhizoctonia solani,* that degrades camalexin through 5’-hydroxylation of the indole ring or through the formation of an oxazoline derivate [39] and the stem rot phytopathogen *Sclerotinia sclerotiorum* which is able to transform camalexin into the glycosylated derivate at N-1 or C-6 of the indole ring [40].

Despite the importance of the root as a plant organ in continuous contact with the rhizosphere pathogens, there is not much information about the antimicrobial compounds that integrate the root exudates. A few reports describe the exudation of secondary metabolites after elicitation of the root with fungal pathogens, and for some of these metabolites antimicrobial activities against a wide range of microorganisms have been shown (reviewed in [1]). A detailed analysis of root exudates in *Arabidopsis* challenged with *Pseudomonas syringae* provides a strong argument for the role of antimicrobial compounds from the root exudates in the plant defence mechanism to this bacterium [41]. Bais *et al*. showed that seven out of eight strains of *P. syringae* are unable to infect *Arabidopsis* and they describe how non-pathogenic strains induce the exudation of more secondary metabolites than non-infected plants or the plants infected with a virulent strain. The bacteriostatic activity of the root exudates elicited by non-pathogenic bacteria was measured and found to be moderate against the seven non-infecting strains. Accordingly, the root exudates elicited by the infecting strain had no significant bacteriostatic activity against any of the *P. syringae* strain. More interestingly the authors identify the possible mechanism of resistance of the infecting strain to the antimicrobial compounds, and they proposed that this strain is able to both block the exudation and partially resists to the antibiotic through its type III secretion system. This work provides an excellent example of the determinant role of some phytoalexins and phytoanticipins in arresting pathogen growth and how some pathogens have evolved to overcome this defence mechanism.

## 3. Use of Phytoanticipins and Phytoalexins as Antibacterial Agents in Human Medicine

Two major circumstances have accentuated research aiming at the discovery of antibacterial agents derived from plant natural products in the last decade. First, nosocomial and community-acquired infections caused by bacteria that are resistant to more than two classes of conventional antibiotics represent an increasingly important public health concern. A reason for the problem of multidrug resistance (MDR) resides on the fact that the current arsenal of antibiotics has been largely designed on limited chemical scaffolds with only few innovations since the 1980s, leaving an opportunity for pathogens to develop and spread antibiotic resistance mechanisms worldwide [42,43]. Second, the high popularity and general acceptance of natural products as tools for disease prevention and health maintenance have made discovery efforts for specific bioactive components from plant extracts surge [44,45], and there are now numerous reports of plant products providing antibiotic activities against a wide variety of pathogenic bacteria. Multiple classes of antibacterial products, including phenolic acids and polyphenols [46], phenanthrenes [47], flavonoids [48], terpenoids [49] have been described and the bioactivities of many more plant products and essential oils are reviewed elsewhere [44,50,51]. Interestingly, at this time, no product has been approved for systemic use to combat bacterial infections, in part because the spectrum of activity or the mode of action of purified components is often very narrow or non specific, respectively. It also has been difficult to isolate specific active components from plant extracts consisting of a mixture of a large number of structurally related compounds with varying degrees of bioactivity or even opposing effects (growth inhibitors *vs* growth stimulants) and even some with cytotoxicity [52]. Most of the bacterial plant pathogens are Gram negative and most of the biologically active purified plant products show low activity against such organisms. Gram positive bacteria are often nevertheless susceptible to plant products and this suggests that the fundamental morphological differences in the cell wall and membrane organization of Gram negative and Gram positive organisms modulate their susceptibility to purified phytoanticipins and phytoalexins. This also suggests that the combine effects of the mixture of natural compounds found *in planta* might be necessary to obtain a synergistic antibacterial activity against Gram negative organisms. Of course, several successful plant pathogens are nevertheless able to circumvent the toxic effects of these plant metabolites.

**Figure 3.**
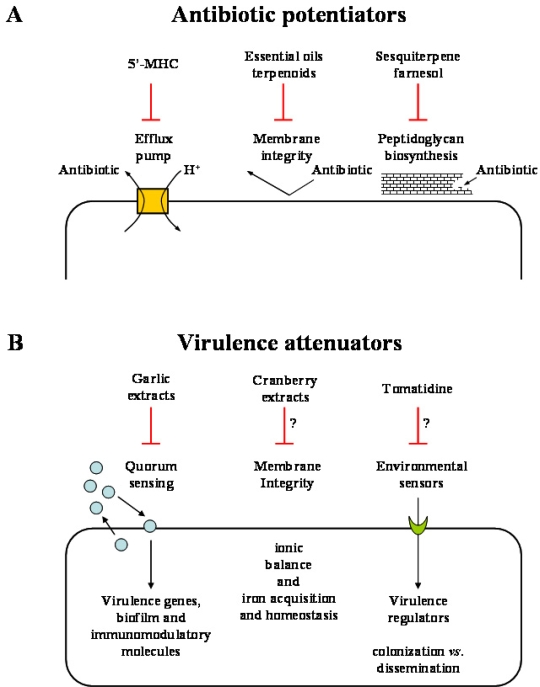
Some examples of plant products defined as “antibiotic potentiators” (A) or “virulence attenuators” (B) could allow the current conventional arsenal of antibiotics to gain back some of the therapeutic applications lost from the spread of MDR and others could assist the host immune system to adequately respond to the pathogen invasion. 5’-MHC, 5’-methoxyhydnocarpin.

Thanks to the persisting efforts of the scientific community, advances in the understanding of the mode of action of bioactive plant products and in the experimental approaches needed to evidence the bioactivities of various plant extracts and individual compounds have allowed identification of interesting leads that enhance the probability of some therapeutic applications. The following discussion provides some original examples of how natural plant products can contribute to enhance the weaponry needed to tackle pathogenic bacteria affecting humans. Currently, two potential applications of phytoanticipins or phytoalexins seem adequate for therapeutic use. Some plant products defined as “antibiotic potentiators” could allow the current conventional arsenal of antibiotics to gain back some of the therapeutic applications lost from the spread of MDR, and others, defined as “virulence attenuators” could assist the host immune system to adequately respond to the pathogen invasion (Figure 3).

Several subtypes of plant products can be considered antibiotic potentiators. The most interesting family, *i.e*., bacterial efflux pump inhibitors, has met with convincing success in demonstrating marked synergy when used in combination with conventional antibiotics against a variety of both Gram positive and Gram negative organisms. The MDR pumps are indeed among the major contributors in the intrinsic resistance of bacteria against a variety of toxic molecules such as alkaloid amphipathic cations, corresponding to many types of secondary metabolites found and produced in plants [53–56].

Among putative efflux pump inhibitors, a catechin (epigallocatechin-gallate) found in green tea extracts abolished tetracycline resistance in staphylococcal isolates expressing TetK, one of the efflux pumps primarily found in Gram positive bacteria [57]. The mode of action of the catechin was attributed to the inhibition of the efflux pump by measuring the relative amount of tetracycline extruded from bacteria in the presence or absence of the catechin. Interaction with efflux pumps was further supported by the lack of minocycline potentiation in the presence of catechin since this semisynthetic tetracycline is known not to be a substrate for efflux pumps. Interestingly however, epigallocatechin-gallate was also found to potentiate the activity of β-lactam antibiotics against methicillin-resistant *Staphylococcus aureus* (MRSA) by a mechanism other than efflux pump inhibition indicating the possible existence of a second mode of action [58]. Experiments from Zhao *et al*. [58] demonstrated binding of the catechin to the bacterial cell wall and hypersensitization of *S. aureus* to high ionic strength and low osmotic pressure. Similarly baicalein, a 5,6,7-trihydroflavone found in thyme extracts, also potentiates the antibacterial activity of tetracyclines and β-lactams against MRSA [59]. The inhibition of efflux pumps was demonstrated by blocking uptake of [^3^H]tetracycline in inverted bacterial membrane vesicles prepared from TetK^+^ *E. coli*.

Interestingly, it exist at least one example of a mixture of an efflux pump inhibitor and a bactericidal compound naturally combined *in planta*. The medicinal plants *Berberis* were shown to produce both the antibacterial alkaloid berberine as well as the NorA efflux pump inhibitor 5’-methoxyhydnocarpin [60]. The single-component NorA pump of *S. aureus* is a chromosomally-encoded multidrug proton-dependent efflux transporter that is a member of the widespread major facilitator superfamily [53]. Against *S. aureus*, the NorA pump inhibitor had no antibacterial activity of its own but substantially potentiated the activity of NorA cationic substrates such as berberine and some fluoroquinolones. The action of 5’-methoxyhydnocarpin on NorA was identified by the inhibition of release of fluorescent berberine or ethidium bromide (another NorA substrate) from drug-preloaded *S. aureus* cells. Inversely, screening for efflux pump inhibitors in plant extracts using bioassays designed to detect synergy with conventional drugs led to the isolation of *N*-*trans*-feruloyl 4’-*O*-methyldopamine from the methanolic extract of *Mirabilis jalapa*. This molecule was able to block NorA and thus significantly improve the activity of norfloxacin against *S. aureus* [61]. Bacterial pump inhibitors discovered from plant sources have recently been reviewed [62,63].

The observed synergy between berberine and 5’-methoxyhydnocarpin and the elucidation of its mode of action toward NorA has triggered the development of screens for identification of plant products that have antibiotic activities against Gram negative bacteria. In an example of such a screen, the utilization of known synthetic inhibitors of Gram negative multidrug resistance pumps has revealed the potential broad spectrum antibacterial activity of rhein, plumbagin, resveratrol, gossypol, coumestrol and berberine [64].

Other plant products considered as antibiotic potentiators include examples of cell wall acting agents and membrane destabilizing agents. A major fraction of essential oils from plant extracts is composed of terpenoids which are defined by an isoprene structure of lipophilic nature. Synergy between major classes of clinically relevant antibiotics and sesquiterpenoids such as farnesol, nerolidol and others has been demonstrated. As opposed to efflux pump inhibition, the mode of action of terpenoids may involve, at least in part, bacterial membrane permeabilization as demonstrated by studying intracellular accumulation of ethidium bromide using flow cytometry [65] and by measuring K^+^ ion leakage [49]. More recently, the sesquiterpene farnesol was shown to drastically increase the susceptibility of MRSA toward β-lactams by specifically inhibiting the recycling of the C_55_ lipid carrier needed in bacterial cell wall peptidoglycan biosynthesis [66].

The attenuation of virulence as opposed to the direct killing of pathogenic bacteria as a strategy to combat infections is an interesting concept. The thought is that antipathogenic molecules that prevent for instance the production of toxins or abolish the ability of bacteria to adapt to the mammalian environment would give a competitive advantage to the host immune system to allow clearance of the infectious organism (Figure 3). It is also anticipated that such virulence attenuators would not affect non-pathogenic bacterial communities or exert a selective pressure for the development of resistance as seen from the pressures exerted by conventional antibiotics that targeted vital bioprocesses in bacteria [67]. One way to interfere with the adaptability of pathogens to the host environment is to block quorum sensing systems that usually synchronize the infection process through the production of small diffusible signalling molecules that accumulate with increasing bacterial cell density [68,69]. Quorum sensing controlled events include the timely induction of a large number of host disabling toxins and hydrolytic enzymes. This bacterial strategy prevents the host to gradually detect the presence of the invader and to adequately build the immune response. Recently, this concept of virulence attenuation was demonstrated by first screening for quorum sensing inhibitory plant extracts [70]. This was achieved by the design of reporter genes fused to quorum sensing-controlled promoters. In such a screen, a garlic extract, but not synthetic allicin, was determined as one of the most potent quorum sensing inhibitors among the samples tested. As such, the garlic extract was further shown to reduce *Pseudomonas aeruginosa* biofilm tolerance to tobramycin treatment. Furthermore, since bacterial biofilm development is an important quorum sensing-mediated process needed for host tissue colonization by pathogens such as *P. aeruginosa* in respiratory infections of cystic fibrosis patients, Bjarnsholt *et al*. [71] tested the prophylactic properties of garlic extracts in a pulmonary mouse model of infection. The garlic extract was administered subcutaneously prior to the instillation of the bacteria in the lungs and treatment was continued until the mice were sacrificed. Results showed that the garlic extract improved clearance of the infecting bacteria and it seemed that not only the garlic extract could modulate bacterial quorum sensing events but also, directly or indirectly, adequately modulate the host inflammatory response. Since the inflammatory response and the infection profile of a quorum sensing mutant is similar to that observed in garlic-treated mice infected with a wild type strain, it is tempting to speculate that the improved host response by the garlic extract is directly mediated by the inhibition of bacterial quorum sensing systems. This hypothesis is further supported by the observation that pseudomonal quorum sensing signalling molecules such as *N*-acyl homoserine lactones can be detected in the sputum of cystic fibrosis patients [72,73] and that such signalling molecules were shown to have immunomodulatory activities [74,75].

As for the use of efflux pump inhibitors in experimental screens to uncover the true antibacterial potential of plant products, new investigational techniques evaluating the specific transcriptional stress responses generated by exposure of bacteria to plant products have recently helped identifying bioactive plant extracts and the putative mode of action of antibacterial compounds. For example, microarray-derived transcriptional analyses confirmed the repression of quorum sensing controlled gene expression in *P. aeruginosa* exposed to garlic extracts [70]. More recently, the transcriptional profiles of *S. aureus* treated with the anthraquinone rhein [76] and the alkaloid berberine [77] were disclosed. Results suggest that rhein perturbs *S. aureus* anaerobic respiration and fermentation [76] and further support the expression of efflux pumps in modulating the susceptibility of *S. aureus* to berberine [77].

Our own transcriptional analyses of bacteria exposed to plant products have also helped understanding their mechanisms of action. For instance, it is well known that the cranberry fruit (*Vaccinium macrocarpon Ait*.) possesses intrinsic antimicrobial properties against many pathogens and that some effects may be linked to its bacterial anti-adhesion activity against pathogens [78]. Other effects however are possibly due to the various acids and phenolics found in this fruit. The mechanisms supporting the diverse effects of cranberry fruit extracts (CFEs) on microbes are poorly understood. We thus recently studied the effect of CFEs on *E. coli* using a DNA array-based approach in an attempt to correlate specific transcriptional signatures and bacterial cell damages [79]. Treatment of *E. coli* with CFEs strongly down-regulated OmpF and overexpressed TolQ and Gad, all involved in membrane functions, maintenance of ionic balance and protection against high-proton-concentration environments [80–82] suggesting important membrane disturbances. The CFEs also strongly down-regulated the expression of several iron-uptake genes (*ent, feo, fep, exb* and others) negatively regulated by the transcriptional repressor Fur to limit accumulation of intracellular iron and to prevent iron-derived oxidative stresses. Accordingly, extracts were also found to up-regulate genes (ferritin, iron superoxide dismutase, fumarase) normally expressed in iron-rich conditions. Such genes are known to be negatively regulated by the small RNA *ryhB* which expression is in turn negatively regulated by Fur [83]. In sum, the effects observed on the transcriptome of *E. coli* exposed to cranberry extracts correlated with known characteristics of cranberry constituents such as condensed tannins (flavonoids) and phenolics that could possibly act as iron chelators. In view of these results, cranberry extracts could potentially lead to the development of agents that perturb bacterial homeostasis.

Some of our recent works also shed light on the bioactivity of tomatidine, the aglycon version of the phytoanticipin tomatine, on *S. aureus* [84]. Although the minimal inhibitory concentration of the compound was high (>128 μg/mL), tomatidine interestingly inhibited hemolysin production by *S. aureus* on blood agar plates. Accordingly, transcriptional analyses of *S. aureus* exposed to tomatidine showed a striking down-regulation of many extracellular toxins, including alpha-hemolysin and delta-hemolysin (*i.e*., RNAIII, the effector molecule of the quorum sensing Agr system), serine proteases, lipases and nucleases. This modulation of gene expression was seen using tomatidine concentrations as low as 1.28 μg/mL and suggests a possible application for tomatidine as a virulence attenuator. Furthermore, we are currently also assessing the anti-virulence activity of tomatidine on *S. aureus* small colony variants (SCV) which are slow-growing respiratory deficient derivatives often found in the lungs of cystic fibrosis patients [85]. We have previously shown that SCV from cystic fibrosis patients have a transcriptional signature of their own that results in expression of virulence factors involved in host tissue colonization, cellular invasion and biofilm formation, which are likely to play a role in chronic infections [86–88]. We found that biofilm production, which was very elevated in SCV compared to prototypical *S. aureus* strains, was specifically inhibited by tomatidine at subminimal inhibitory concentrations [88]. These results indicate that tomatidine has an overall effect on virulence determinants in *S. aureus* and may eventually provide a new avenue for the management of both acute and chronic lung infections in cystic fibrosis patients. We have shown here that various types of plant products may be used in combination with conventional antibiotics to achieve antibacterial synergy. The future of phytoalexins and phytoanticipins as “antibiotic potentiators” or “virulence attenuators” for use in human medicine is thus promising.

## 4. Biotechnological Applications of Phytoanticipins and Phytoalexins in Phytoprotection

The ultimate objective of investigations studying the relevance of phytoanticipins and phytoalexins in the plant defence response is to develop biotechnological applications in crop protection. Some of the above described phytoalexins provide a potentially interesting weapon to be used in agricultural techniques. The use of phytoalexins and phytoanticipins in phytoprotection however entails some disadvantages that have to be overcome.

The use of the phytoalexin itself as a phytoprotectant presumes that the molecule is only toxic against pathogenic agents. This is very often not the case and several antimicrobial compounds have shown quite unspecific toxicities. Moreover, the synthesis or isolation of phytoalexins is very expensive compared to commercial fungicidal molecules [2].

An alternative approach would be the production of plants that express a higher quantity of phytoalexins either by spraying with phytoalexin elicitors, by pre-immunization through a non-pathogen inoculation, or by genetic transformation. One problem of the first approach is that plants continuously elicited to produce phytoalexins results in stunted plants which produce a yield as poor as or poorer than the infected plants [2]. Thus, an adequate regulation of the production of phytoalexins could help resolving the problem. Indeed, transfer of genes involved in the synthesis of phytoalexins to yield more resistant crops has been proven successful in the case of tobacco plants in which transformation with stilbene synthase, involved in resveratrol synthesis, provided plant resistance to *B. cinerea* infection [89].

An additional problem to the use of phytoanticipins and phytoalexins in crop protection is the capacity of some pathogens to detoxify phytoalexins into less toxic compounds, or furthermore into compounds that can suppress the establishment of a defence response. This activity can be so important into the pathogenesis process that it can determine the disease severity of some fungi [90]. The discovering and understanding of inhibitors of phytoalexin-detoxifying enzymes is crucial to overcome this problem, and it opens a wide variety of biotechnological applications for a new generation of chemicals called paldoxins (from phytoalexins detoxification inhibitor) designed to provide sustainable treatments of agricultural crops [91]. The use of paldoxin will allow the accumulation of the natural defence of the plant in an environmental safer way, since selective inhibitors are less likely to affect non-targeted organisms.

The crucifer phytoalexin brassinin is detoxified by *L. maculans* and *S. sclerotiorum*. To date, all the potential paldoxins found against *S. sclerotiorum* seem to be metabolized by the fungus, but results seem more promising for *L. maculans*. Screening of a potential paldoxin library identified four compounds that decreased the rate of brassinin detoxification by *L. maculans* and these compounds are *N’*-methylbrassinin, naphthyl dithiocarbamate, indolyl dithiocarbonate and phenyl dithiocarbazate. *N’*-methylbrassinin displayed a higher antifugal activity relative to brassinin and thus represents a potential paldoxin that could be used in crop protection. Other compounds probed to be better inhibitors of fungal growth, but they were also targets of degradation [27]. In a more recent work, new paldoxins were designed based on the camalexin scaffold because *L. maculans* is unable to metabolize it [92]. As a result, an even better inhibitor of brassinin oxidase was discovered but unfortunately, this compound also induced fungal pathways protecting the microorganism against oxidative stresses and brassinin toxicity. Therefore, although paldoxins seem to represent promising chemicals to control pathogen infections, a careful analysis of the effect of the molecules in both plant and pathogen metabolisms is essential before use of such antimicrobials in crop protection.

## 5. Conclusions

The numerous examples of plant secondary metabolites (phytoalexins and phytoanticipins) reviewed here demonstrate that they constitute an important mechanism to stop spreading of phytopathogens *in planta*, both by acting as antimicrobials themselves or as elicitors of other defence responses. More interestingly, some examples described here show that phytoalexins and phytoanticipins are also active against clinically-relevant pathogens and their use as “antibiotic potentiators” or “virulence attenuators” for the control of infectious diseases in humans is promising. The progressing threat of MDR for public health and the incessant need for crop protection strengthen the importance of the research activities aiming at the isolation and characterization of plant secondary metabolites and the understanding of the mechanisms involved in the natural defences of plants against microbial aggressors.

## References

[1] Bais HP Weir TL Perry LG Gilroy S Vivanco JM The role of root exudates in rhizosphere interactions with plants and other organisms Annu. Rev. Plant Biol 2006 57 233 266 16669762 10.1146/annurev.arplant.57.032905.105159

[2] Grayer RJ Kokubun T Plant-fungal interactions: The search for phytoalexins and other antifungal compounds from higher plants Phytochemistry 2001 56 253 263 11243452 10.1016/s0031-9422(00)00450-7

[3] Jones JD Dangl JL The plant immune system Nature 2006 444 323 329 17108957 10.1038/nature05286

[4] Lindsay WP Lamb CJ Dixon RA Microbial recognition and activation of plant defense systems Trends Microbiol 1993 1 181 187 8143136 10.1016/0966-842x(93)90088-9

[5] Bednarek P Osbourn A Plant-microbe interactions: Chemical diversity in plant defense Science 2009 324 746 748 19423814 10.1126/science.1171661

[6] Müller KO Börger H Experimentelle Untersuchungen über die *Phytophtora*-Resistenz, Kartoffel [In German] Arb. Biol. Reichsanstalt. Landw. Forstw. Berlin 1940 23 189 231

[7] VanEtten HD Mansfield JW Bailey JA Farmer EE Two classes of plant antibiotics: Phytoalexins versus phytoanticipins Plant Cell 1994 6 1191 1192 12244269 10.1105/tpc.6.9.1191 PMC160512

[8] Osbourn AE Preformed antimicrobial compounds and plant defense against fungal attack Plant Cell 1996 8 1821 1831 12239364 10.1105/tpc.8.10.1821 PMC161317

[9] Osbourn AE Saponins in cereals Phytochemistry 2003 62 1 4 12475612 10.1016/s0031-9422(02)00393-x

[10] Morrissey JP Osbourn AE Fungal resistance to plant antibiotics as a mechanism of pathogenesis Microbiol. Mol. Biol. Rev 1999 63 708 724 10477313 10.1128/mmbr.63.3.708-724.1999 PMC103751

[11] Osbourn AE Clarke BR Lunness P Scott PR Daniels MJ An oat species lacking avenacin is susceptible to infection by *Gaeumannomyces graminis* var. *tritici* Physiol. Mol. Plant Pathol 1994 45 457 467

[12] Bowyer P Clarke BR Lunness P Daniels MJ Osbourn A Host range of a plant pathogenic fungus determined by a saponin detoxifying enzyme Science 1995 267 371 374 7824933 10.1126/science.7824933

[13] Papadopoulou K Melton RE Legget M Daniels MJ Osbourn AE Compromised disease resistance in saponin deficient plants Proc. Natl. Acad. Sci. USA 1999 96 12923 12928 10536024 10.1073/pnas.96.22.12923 PMC23166

[14] Mylona P Owatworakit A Papadopoulou K Jenner H Qin B Findlay K Hill L Qi X Bakht S Melton R Osbourn A Sad3 and sad4 are required for saponin biosynthesis and root development in oat Plant Cell 2008 20 201 212 18203919 10.1105/tpc.107.056531 PMC2254932

[15] Martín-Hernandez AM Dufresne M Hugouvieux V Melton R Osbourn A Effects of targeted replacement of the tomatinase gene on the interaction of *Septoria lycopersici* with tomato plants Mol. Plant Microbe Interact 2000 13 1301 1311 11106022 10.1094/MPMI.2000.13.12.1301

[16] Bouarab K Melton R Peart J Baulcombe D Osbourn A Nature 2002 418 889 892 12192413 10.1038/nature00950

[17] Maor R Shirasu K The arms race continues: Battle strategies between plants and fungal pathogens Curr. Opin. Microbiol 2005 8 399 404 15996507 10.1016/j.mib.2005.06.008

[18] Valle T Lopez JL Hernandez JM Corchete P Antifungal activity of scopoletin and its differential accumulation in *Ulmus pumila* and *Ulmus campestris* cell suspension cultures infected with *Ophiostoma ulmi* spores Plant Sci 1997 125 97 101

[19] Chong J Baltz R Schmitt C Beffa R Fritig B Saindrenan P Downregulation of a pathogen-responsive tobacco UDP-Glc:phenylpropanoid glucosyltransferase reduces scopoletin glucoside accumulation, enhances oxidative stress, and weakens virus resistance Plant Cell 2002 14 1093 1107 12034899 10.1105/tpc.010436 PMC150609

[20] Matros M Mock HP Ectopic expression of a UDP-glucose:phenylpropanoid glucosyl-transferase leads to increased resistance of transgenic tobacco plants against infection with Potato Virus Y Plant Cell Physiol 2004 45 1185 1193 15509841 10.1093/pcp/pch140

[21] Costet L Fritig B Kauffmann S Scopoletin expression in elicitor-treated and tobacco mosaic virus-infected tobacco plants Physiologia Plantarum 2002 115 228 235 12060240 10.1034/j.1399-3054.2002.1150208.x

[22] Chong J Baltz R Fritig B Saindrenan P An early salicylic acid-, pathogen- and elicitor-inducible tobacco glucosyltransferase: Role in compartmentalization of phenolics and H_2_O_2_ metabolism FEBS Lett 1999 458 204 208 10481066 10.1016/s0014-5793(99)01154-0

[23] Rakwal R Agrawal GK Yonekura M Kodama O Naringenin 7-*O*-methyltransferase involved in the biosynthesis of the flavanone phytoalexin sakuranetin from rice (*Oryza sativa* L.) Plant Science 2000 155 213 221 10814825 10.1016/s0168-9452(00)00223-5

[24] Cartwright DW Langcake P Pryce RJ Leworthy DP Ride JP Isolation and characterization of two phytoalexins from rice as momilactones A and B Phytochemistry 1981 20 535 537

[25] Dillon VM Overton J Grayer RJ Harborne JB Difference in phytoalexins response among rice cultivars of different resistance to blast Phytochemistry 1997 44 599 603

[26] Rodrigues FA McNally DJ Datnoff LE Jones JB Labbé C Benhamou N Menzies JG Belanger RR Silicon enhances the accumulation of diterpenoid phytoalexins in rice: A potential mechanism for blast resistance Phytopathology 2004 94 177 183 18943541 10.1094/PHYTO.2004.94.2.177

[27] Pedras MS The chemical ecology of crucifers and their fungal pathogens: Boosting plant defenses and inhibiting pathogen invasion Chem. Rec 2008 8 109 115 18383155 10.1002/tcr.20140

[28] Pedras MSC Zheng QA Sarma-Mamillapalle VK The phytoalexins from Brassicaceae: Structure, biological activity, synthesis and biosynthesis Nat. Prod. Commun 2007 2 319 330

[29] Bendarek P Pislewska-Bednarek M Svatos A Schneider B Doubsky J Mansurova M Humphry M Consonni C Panstruga R Sanchez-Vallet A Molina A Schulze-Lefert P A glucosinolate metabolism pathway in living plant cells mediates broad-spectrum antifungal defenses Science 2009 323 101 106 19095900 10.1126/science.1163732

[30] Clay NK Adio AM Denoux C Jander G Ausubel FM Glucosinolate metabolites required for an Arabidopsis innate immune response Science 2009 323 95 101 19095898 10.1126/science.1164627 PMC2630859

[31] Glawischnig E Camalexin Phytochemistry 2007 68 401 406 17217970 10.1016/j.phytochem.2006.12.005

[32] Schuhegger R Rauhut T Glawischnig E Regulatory variability of camalexin biosynthesis J. Plant Physiol 2007 164 636 644 16769150 10.1016/j.jplph.2006.04.012

[33] Kliebenstein DJ Rowe HC Denby KJ Secondary metabolites influence *Arabidopsis/Botrytis* interactions: Variation in host production and pathogen sensitivity Plant J 2005 44 25 36 16167893 10.1111/j.1365-313X.2005.02508.x

[34] Schuhegger R Nafisi M Mansourova M Petersen BL Olsen CE Svatos A Halkier BA Glawischnig E CYP71B15 (PAD3) catalyzes the final step in camalexin biosynthesis Plant Physiol 2006 141 1248 1254 16766671 10.1104/pp.106.082024 PMC1533948

[35] Rogers EE Glazebrook J Ausubel FM Mode of action of the *Arabidopsis thaliana* phytoalexins camalexin and its role in *Arabidopsis*-pathogen interactions Mol. Plant Microb. Interact 1996 9 748 757 10.1094/mpmi-9-0748 8870273

[36] Thomma BP Nelissen I Eggermont K Broekaert WF Deficiency in phytoalexin production causes enhanced susceptibility of *Arabidopsis thaliana* to the fungus *Alternaria brassicola* Plant J 1999 19 163 171 10476063 10.1046/j.1365-313x.1999.00513.x

[37] Chassot C Buchala A Schoonbeek HJ Métraux JP Lamotte O Wounding of Arabidopsis leaves causes a powerful but transient protection against *Botrytis* infection Plant J 2008 55 555 567 18452590 10.1111/j.1365-313X.2008.03540.x

[38] Stefanato FL Abou-Mansour E Buchala A Kretschmer M Mosbach A Bochet CG Métraux JP Schoonbeek HJ The ABC transporter BcatrB from *Botrytis cinerea* exports camalexin and is a virulence factor on *Arabidopsis thaliana* Plant J 2009 58 499 510 19154205 10.1111/j.1365-313X.2009.03794.x

[39] Pedras MSC Khan AQ Biotransformation of the phytoalexins camalexin by the phytopathogen *Rhizoctonia solani* Phytochemistry 2000 53 59 69 10656409 10.1016/s0031-9422(99)00479-3

[40] Pedras MSC Ahiahonu PW Probing the phytopathogenic stem rot fungus with phytoalexins and analogues: Unprecedented glucosylation of camalexin and 6-methoxycamalexin Bioorg. Med. Chem 2002 10 3307 3312 12150877 10.1016/s0968-0896(02)00208-0

[41] Bais HP Prithiviraj B Jha AK Ausubel FM Vivanco JM Mediation of pathogen resistance by exudation of antimicrobial from roots Nature 2005 434 217 221 15759001 10.1038/nature03356

[42] Shah PM The need for new therapeutic agents: What is in the pipeline Clin Microbiol Infect 2005 11 Suppl. 3 36 42 15900655 10.1111/j.1469-0691.2005.01141.x

[43] Talbot GH Bradley J Edwards JE Jr Gilbert D Scheld M Bartlett JG Bad bugs need drugs: An update on the development pipeline from the antimicrobial availability task force of the infectious diseases society of america Clin. Infect. Dis 2006 42 657 668 16447111 10.1086/499819

[44] Cowan MM Plant products as antimicrobial agents Clin. Microbiol. Rev 1999 12 564 582 10515903 10.1128/cmr.12.4.564 PMC88925

[45] Pauli GF Case RJ Inui T Wang Y Cho S Fischer HH Franzblau SG New perspectives on natural products in TB drug research Life Sci 2005 78 485 494 16243360 10.1016/j.lfs.2005.09.004

[46] Taguri T Tanaka T Kouno I Antibacterial spectrum of plant polyphenols and extracts depending upon hydrogyphenyl structure Biol. Pharm. Bull 2006 29 2226 2235 17077519 10.1248/bpb.29.2226

[47] Kovacs A Vasas A Hohmann J Natural phenanthrenes and their biological activity Phytochem 2008 69 1084 1110 10.1016/j.phytochem.2007.12.005 18243254

[48] Cushnie TTP Lamb AJ Antimicrobial activity of flavonoids Int. J. Antimicrob. Agents 2005 26 343 356 16323269 10.1016/j.ijantimicag.2005.09.002 PMC7127073

[49] Inou Y Shiraishi A Hada T Hirose K Hamashima H Shimada J The antibacterial effects of terpene alcohols on *Staphylococcus aureus* and their mode of action. *FEMS Microbiol* Lett 2004 237 325 331 10.1016/j.femsle.2004.06.049 15321680

[50] Kalemba D Kunicka A Antibacterial and antifungal properties of essential oils Curr. Medic. Chem 2003 10 813 829 10.2174/0929867033457719 12678685

[51] Rios JL Recio MC Medicinal plants and antimicrobial activity J. Ethnopharm 2005 100 80 84 10.1016/j.jep.2005.04.025 15964727

[52] Jaki BU Franzblau SG Chadwick LR Lankin DC Zhang F Wang Y Pauli GF Purity-activity relationships of natural products: The case of anti-TB active ursolic acid J. Nat. Prod 2008 71 1742 1748 18798682 10.1021/np800329j

[53] Li X-Z Nikaido H Efflux-mediated drug resistance in bacteria Drugs 2004 64 159 204 14717618 10.2165/00003495-200464020-00004

[54] Pagès J-M Masi M Barbe J Inhibitors of efflux pumps in gram-negative bacteria Trends Mol. Med 2005 11 382 389 15996519 10.1016/j.molmed.2005.06.006

[55] Poole K Efflux-mediated antimicrobial resistance J. Antimicrob. Chemother 2005 56 20 51 15914491 10.1093/jac/dki171

[56] Lewis K In search of natural substrates and inhibitors of MDR pumps J. Mol. Microbiol. Biotechnol 2001 3 247 254 11321580

[57] Roccaro AS Blanco AR Giuliano F Rusciano D Enea V Epigallocatechin-gallate enhances the activity of tetracycline in Staphylococci by inhibiting its efflux from bacterial cells Antimicrob. Agents Chemother 2004 48 1968 1973 15155186 10.1128/AAC.48.6.1968-1973.2004 PMC415601

[58] Zhao W-H Hu Z-Q Okubo S Hara Y Shimamura T Mechanism of synergy between epigallocatechin gallate and β-lactams against methicillin-resistant *Staphylococcus aureus* Antimicrob. Agents Chemother 2001 45 1737 1742 11353619 10.1128/AAC.45.6.1737-1742.2001 PMC90539

[59] Fujita M Shiota S Kuroda T Hatano T Yoshida T Mizushima T Tsuchiya T Remarkable synergies between baicalein and tetracycline, and baicalein and β-lactams against methicillin-resistant *Staphylococcus aureus* Microbiol. Immunol 2005 49 391 396 15840965 10.1111/j.1348-0421.2005.tb03732.x

[60] Stermitz FR Lorenz P Tawara JN Zenewicz LA Lewis K Synergy in a medicinal plant: Antimicrobial action of bergerine potentiated by 5’-methoxyhydnocarpin, a multidrug pump inhibitor Proc. Natl. Acad. Sci. USA 2000 97 1433 1437 10677479 10.1073/pnas.030540597 PMC26451

[61] Michalet S Cartier G David B Mariotte A-M Dijoux-franca M-G Kaatz GW Stavri M Gibbons S N-Caffeoylphenalkylamide derivatives as bacterial efflux pump inhibitors Bioorg. Med. Chem. Lett 2007 17 1755 1758 17275293 10.1016/j.bmcl.2006.12.059

[62] Gibbons S Phytochemicals for bacterial resistance-strengths, weaknesses and opportunities Planta Med 2008 74 594 602 18446673 10.1055/s-2008-1074518

[63] Stavri M Piddock LJV Gibbons S Bacterial efflux pump inhibitors from natural sources J. Antimicrob. Chemother 2007 59 1247 1260 17145734 10.1093/jac/dkl460

[64] Tegos G Stermitz FR Lomovskaya O Lewis K Multidrug pump inhibitors uncover remarkable activity of plant antimicrobials Antimicrob. Agents Chemother 2002 46 3133 3141 12234835 10.1128/AAC.46.10.3133-3141.2002 PMC128777

[65] Brehm-Stecher BF Johnson EA Sensitization of *Staphylococcus aureus* and *Escherichia coli* to antibiotics by the sesquiterpenoids nerolidol, farnesol, bisabolol, and apritone Antimicrob. Agents Chemother 2003 47 3357 3360 14506058 10.1128/AAC.47.10.3357-3360.2003 PMC201169

[66] Kuroda M Nagasaki S Ohta T Sesquiterpene farnesol inhibits recycling of the C55 lipid carrier of the murein monomer precursor contributing to increased susceptibility to β-lactams in methicillin-resistant *Staphylococcus aureus* J Antimicrob Chemother 2007 59 425 432 17242033 10.1093/jac/dkl519

[67] Bjarnsholt T Givskov M Quorum-sensing blockade as a strategy for enhancing host defences against bacterial pathogens Phil. Trans. R. Soc. B 2007 362 1213 1222 17360273 10.1098/rstb.2007.2046 PMC2435584

[68] Fuqua C Parsek MR Greenberg EP Regulation of gene expression by cell-to-cell communication: Acyl-homoserine lactone quorum sensing Ann. Rev. Genet 2001 35 439 468 11700290 10.1146/annurev.genet.35.102401.090913

[69] Novick RP Geisinger E Quorum sensing in Staphylococci Annu. Rev. Genet 2008 42 541 564 18713030 10.1146/annurev.genet.42.110807.091640

[70] Rasmussen TB Bjarnsholt T Skindersoe ME Hentzer M Kristoffersen P Köte M Nielsen J Eberl L Bivskov M Screening for quorum-sensing inhibitors (QSI) by use of a novel genetic system, the QSI selector J. Bacteriol 2005 187 1799 1814 15716452 10.1128/JB.187.5.1799-1814.2005 PMC1063990

[71] Bjarnsholt T Jensen PO Rasmussen TB Christophersen L Calum H Hentzer M Hougen H-P Rygaard J Moser C Eberl L Hoiby N Givskov M Garlic blocks quorum sensing and promotes rapid clearing of pulmonary *Pseudomonas aeruginosa* infections Microbiol 2005 151 3873 3880 10.1099/mic.0.27955-0 16339933

[72] Collier DN Anderson L McKnight SL Noah TL Knowles M Boucher R Schwab U Gilligan P Pesci EC A bacterial cell to cell signal in the lungs of cystic fibrosis patients FEMS Microbiol. Lett 2002 215 41 46 12393198 10.1111/j.1574-6968.2002.tb11367.x

[73] Erickson DL Endersby R Kirkham A Stuber K Vollman DD Rabin HR Mitchell I Storey DG *Pseudomonas aeruginosa* quorum-sensing systems may control virulence factor expression in the lungs of patients with cystic fibrosis Infect. Immun 2002 70 1783 1790 11895939 10.1128/IAI.70.4.1783-1790.2002 PMC127834

[74] Smith RS Harris SG Phipps R Iglewski B The *Pseudomonas aeruginosa* quorum-sensing molecule N-(3-oxododecanoyl) homoserine lactone contributes to virulence and induces inflammation *in vivo* J Bacteriol 2002 184 1132 1139 11807074 10.1128/jb.184.4.1132-1139.2002 PMC134808

[75] Telford G Wheeler D Williams P Tomkins PT Appleby P Sewell H Bycroft BW Pritchard DI The *Pseudomonas aeruginosa* quorum-sensing signal molecule N-(3-oxododecanoyl)-L-homoserine lactone has immunomodulatory activity Infect. Immun 1998 66 36 42 9423836 10.1128/iai.66.1.36-42.1998 PMC107855

[76] Yu L Xiang H Fan J Wang D Yang F Guo N Jin Q Deng X Global transcriptional response of *Staphylococcus aureus* to rhein, a natural plant product J. Biotechnol 2008 135 304 308 18514345 10.1016/j.jbiotec.2008.04.010

[77] Wang D Yu L Xiang H Fan J He L Guo N Feng H Deng X Global transcriptional profiles of *Staphylococcus aureus* treated with berberine chloride FEMS Microbiol. Lett 2008 279 217 225 18179590 10.1111/j.1574-6968.2007.01031.x

[78] Puupponen-Pimiä R Nohynek L Alakomi H-L Oksman-Caldentey K-M Bioactive berry compounds–Novel tools against human pathogens Appl. Microbiol. Biotechnol 2005 67 8 18 15578177 10.1007/s00253-004-1817-x

[79] Gattuso M Malouin F Rempel H Diarra MS Transcriptomic analysis of *Escherichia coli* exposed to cranberry extracts Joint Meeting of 48th Annual Interscience Conference on Antimicrobial Agents and Chemotherapy (ICAAC) and 46th Annual Meeting of the Infectious Diseases Society of America Washington, DC, USA October 25–28, 2008 C1 1944

[80] Germon P Ray M-C Vianney A Lazzaroni JC Energy-dependent conformational change in the TolA protein of *Escherichia coli* involves its N-terminal domain, TolQ, and TolR J. Bacteriol 2001 183 4110 4114 11418549 10.1128/JB.183.14.4110-4114.2001 PMC95298

[81] Pagès J-M James CE Winterhalter M The porin and the permeating antibiotic: A selective diffusion barrier in gram-negative bacteria Nat. Rev 2008 6 893 903 10.1038/nrmicro1994 18997824

[82] Tramonti A de Canio M Delany I Scarlato V de Biase D Mechanisms of transcription activation exerted by GadX and GadW at the *gadA* and *gadBC* gene promoters of the glutamate-based acid resistance system in *Echerichia coli* J. Bacteriol 2006 188 8118 8127 16980449 10.1128/JB.01044-06 PMC1698215

[83] Massé E Gottesman S A small RNA regulates the expression of genes involved in iron metabolism in *Escherichia coli* Proc. Natl. Acad. Sci. USA 2002 99 4620 4625 11917098 10.1073/pnas.032066599 PMC123697

[84] Bouarab K El Oirdi M Gattuso M Moisan H Malouin F Plant stress response agents affect *Staphylococcus aureus* virulence genes ICAAC Chicago, IL, USA September 17–20, 2007 Abstract C1–1483.1.

[85] Proctor RA von Eiff C Kahl BC Becker K McNamara P Herrmann M Peters G Small colony variants: A pathogenic form of bacteria that facilitates persistent and recurrent infections Nat. Rev. Microbiol 2006 4 295 305 16541137 10.1038/nrmicro1384

[86] Moisan H Brouillette E Jacob CL Begin PL Michaud S Malouin F The transcription of virulence factors in *Staphylococcus aureus* small colony variants isolated from cystic fibrosis patients is influenced by SigB J. Bacteriol 2006 188 64 76 16352822 10.1128/JB.188.1.64-76.2006 PMC1317593

[87] Mitchell G Lamontagne CA Brouillette É Grondin G Talbot BG Grandbois M Malouin F *Staphylococcus aureus* SigB activity promotes a strong fibronectin-bacterium interaction which may sustain host tissue colonization by small-colony variants isolated from cystic fibrosis patients Mol. Microbiol 2008 70 1540 1555 19007412 10.1111/j.1365-2958.2008.06511.x

[88] Mitchell G Gattuso M Bouarab K Malouin F Tomatidine affects virulence regulators of prototypical *Staphylococcus aureus* and small colony variants of cystic fibrosis patients ICAAC San Francisco, CA, USA September 12–15, 2009 Abstract C1–1341.

[89] Hain R Reif HJ Krause E Langebartels R Kindl H Vernau B Weise W Schmatzer E Schreier PH Disease resistance results from foreign phytoalexins expression in a novel plant Nature 1993 361 153 156 8421520 10.1038/361153a0

[90] VanEtten H Temporini E Wasmann C Phytoalexin (and phytoanticipin) tolerance as a virulence trait: Why is it not required by all pathogens? Physiol. Mol. Plant Pathol 2001 59 83 93

[91] Pedras MSC Minic Z Jha M Brassisin oxidase, a fungal detoxifying enzyme to overcome a plant defense-Purification, characterization and inhibition FEBS J 2008 275 3691 3705 18549452 10.1111/j.1742-4658.2008.06513.x

[92] Pedras MSC Minic Z Sarma-Mamillapalle VK Synthetic inhibitors of the fungal detoxifying enzyme brassinin oxidase based on the phytoalexins camalexin scaffold J. Agric. Food Chem 2009 57 2429 2435 19243099 10.1021/jf803666s

